# RACE1, a Japanese *Blumeria graminis* f. sp. *hordei* isolate, is capable of overcoming partially *mlo*-mediated penetration resistance in barley in an allele-specific manner

**DOI:** 10.1371/journal.pone.0256574

**Published:** 2021-08-23

**Authors:** Takashi Yaeno, Miki Wahara, Mai Nagano, Hikaru Wanezaki, Hirotaka Toda, Hiroshi Inoue, Ayaka Eishima, Masamichi Nishiguchi, Hiroshi Hisano, Kappei Kobayashi, Kazuhiro Sato, Naoto Yamaoka

**Affiliations:** 1 Department of Agriculture, Ehime University, Tarumi, Matsuyama, Japan; 2 Research Unit for Citromics, Ehime University, Tarumi, Matsuyama, Ehime, Japan; 3 Institute of Plant Science and Resources, Okayama University, Chuo, Kurashiki, Okayama, Japan; University of Nebraska-Lincoln, UNITED STATES

## Abstract

Loss-of-function mutation of the *MILDEW RESISTANCE LOCUS O* (*Mlo*) gene confers durable and broad-spectrum resistance to powdery mildew fungi in various plants, including barley. In combination with the intracellular nucleotide-binding domain and leucine-rich repeat receptor (*NLR*) genes, which confer the race-specific resistance, the *mlo* alleles have long been used in barley breeding as genetic resources that confer robust non-race-specific resistance. However, a Japanese *Blumeria graminis* f. sp. *hordei* isolate, RACE1, has been reported to have the potential to overcome partially the *mlo*-mediated penetration resistance, although this is yet uncertain because the putative effects of *NLR* genes in the tested accessions have not been ruled out. In this study, we examined the reproducibility of the earlier report and found that the infectious ability of RACE1, which partially overcomes the *mlo*-mediated resistance, is only exerted in the absence of *NLR* genes recognizing RACE1. Furthermore, using the transient-induced gene silencing technique, we demonstrated that RACE1 can partially overcome the resistance in the host cells with suppressed *MLO* expression but not in plants possessing the null mutant allele *mlo*-5.

## Introduction

Powdery mildew is one of the most widespread diseases of higher plants that is caused by obligate biotrophic ascomycete fungi of the order Erysiphales [1]. Approximately 900 species can infect more than 10,000 plant species [2]. The disease causes significant harvest losses in many crop plants such as barley, wheat, tomato, and cucumber [3,4]. As a reasonable strategy to reduce agricultural costs, breeding of disease resistant crops is performed by introgression of resistance (*R*) genes. Most *R* genes encode the intracellular nucleotide-binding domain and leucine-rich repeat receptors (NLRs) which recognize either the structure of effector proteins secreted from pathogens or altered host cellular responses by effector proteins. The effectors recognized by NLRs are referred to as avirulence (Avr) proteins [5]. The NLR-mediated recognition triggers strong immune responses and frequently results in plant cell death, also known as hypersensitive responses (HR). The responses are especially crucial for defense against obligate biotrophic pathogens. In the interaction between barley and powdery mildew pathogen *Blumeria graminis* f. sp. *hordei* (*Bgh*), the race-specific resistances based on the gene-for-gene manner are conferred by several *NLR* loci. The *Mla* (*MILDEW RESISTANCE LOCUS A*) locus, which is the most well-studied of these, is located on the short arm of chromosome 1H and the numerous alleles have been characterized from many accessions [6–9]. Although not all of the *Avr* genes corresponding to *Mla* alleles have been identified in *Bgh* [10–12], the large number of *Mla* alleles suggests rapid coevolution, and therefore the gene-for-gene resistance conferred by the *Mla* genes is thought to be overcome relatively rapidly by the evolution of Avr effectors [13]. For sustainable resistance breeding, non-race-specific resistance genes are required.

In addition to suppressing the immune responses, an obligate biotrophic fungus such as *Bgh* needs to rely on sustained compatibility with the host plant. *Bgh* penetrates and forms a haustorium in normally viable host epidermal cells and has to absorb nutrients that continue to be biosynthesized in abundance. If the host’s genes, so-called susceptibility (*S*) genes, which are involved in the negative regulation of defenses or necessary to achieve favorable conditions for infection, are lost in function, *Bgh* is unable to establish a compatible interaction. Unlike the *R* gene conferring dominantly-inherited resistance, in most cases, loss of function mutations in the *S* gene confers durable, broad spectrum and recessively-inherited resistance to different races of a pathogen. For example, a recessive mutation in the *Mlo* (*MILDEW RESISTANCE LOCUS O*) gene, which is located on barley chromosome 4H, prevents the penetration of *Bgh* in a race-independent manner. The first *mlo* mutant was found in a barley mutant population in the 1940s, and many alleles have now been identified from accessions around the world and utilized as breeding materials [14]. The *MLO* genes are conserved throughout the plant kingdom and exist as a multicopy gene family in higher plants. Therefore, they have also been utilized as resistance genetic resources in many crops [15–18], and in recent years, the *mlo* mutant of wheat has been not only generated via the transgenic CRISPR/Cas approach, but also non-transgenically created by TILLING [19–21]. In Arabidopsis, among the 15 *MLO* orthologues, the *mlo2* mutation confers partial resistance to the adapted powdery mildew fungus *Golovinomyces orontii* and reduces the penetration rate by about 50%. Besides, the *mlo2 mlo6 mlo12* triple mutant shows complete resistance to the fungus [22]. The *Mlo* gene of barley encodes a seven-transmembrane domain protein [23]. MLO proteins accumulate at the site of penetration from the appressorium of *Bgh* [24]. The C-terminal cytoplasmic tail of MLO is thought to regulate the penetration resistance by interacting with the calcium sensor protein calmodulin, but the detailed molecular mechanism is still unclear [25].

In 1995, it was reported that a Japanese *Bgh* isolate, RACE1, could partially overcome the penetration resistance conferred by the *mlo* mutation [26]. However, at that time, it has been difficult to interpret the results accurately because the *mlo* mutant alleles isolated from different accessions were used and it was not clear what genetic background they had or whether they possessed some *NLR* gene or not. Therefore, in this study, we investigated whether the report could be reproduced, taking into account the *NLR* genes possessed by each accession and the *Avr* genes of RACE1. Furthermore, we confirmed that RACE1 could overcome the *mlo*-mediated penetration resistance in host cells in which the expression of *MLO* was suppressed.

## Materials and methods

### Plant materials and pathogen inoculation

Barley *mlo* accessions that had been provided by Prof. J. H. Jørgensen in 1994 and stocked in the Institute of Plant Science and Resources, Okayama University were used for the analysis of *Bgh* morphogenesis. The barley accessions ‘Morex’, ‘Golden Promise’ and ‘Haruna Nijo’ were provided by the institute with support in part by the National Bio-Resource Project of the MEXT, Japan. Seedlings were grown on fine-grained vermiculite supplemented with 300-fold diluted HYPONeX (N:P:K = 6:10:5, HYPONeX Japan, Osaka, Japan) in a growth chamber (NK system Biotron model LH-200-RD, Nippon Medical & Chemical Instruments, Osaka, Japan) at 20°C in continuous fluorescent light. Six- or seven-day-old seedlings were used in the experiments. *Bgh* isolates OU14 (*AVRa1*, *AVRa6*, *AVRa7*, *AVRa13*, and *AVRa15*) and RACE1 (*AVRa6*, *AVRa7*, *AVRa8*, *AVRa13*, and *AVRa15*) [10] were maintained on the cultivars ‘Haruna Nijo’ and ‘Kobinkatagi’, respectively, in the growth chamber (NK system Biotron model LH-200-RD) at 20°C in continuous fluorescent light. In this study, the isolate formerly known as “Race I” is described as RACE1 as the genomically analysed culture strain according to Frantzeskakis et al. [27]. Leaves were inoculated with *Bgh* as described by Sugai et al. [28]. After inoculation, the seedlings were incubated in the chamber (NK system LH-60FL3-DT, Nippon Medical & Chemical Instruments, Osaka, Japan).

### Analysis of the morphogenesis of *Bgh* isolates

The primary leaf of each 7-day-old accession was inoculated with *Bgh* and stained with trypan blue 4 days later. Trypan blue staining was performed as described by Yaeno and Iba [29], except that a mixture of staining solution and ethanol at a ratio of 1:1 was used. An optical microscope (BX53, Olympus, Tokyo, Japan) was used for observation. The proportion of each morphology of OU14 and RACE1 isolates in each accession was calculated by counting the morphology of the conidia in situations where only one conidium came into contact with one epidermal cell. Approximately 100 to 200 conidia per plant of each accession were examined.

### Transient induced gene silencing assay

To suppress the expression of the *Mlo* gene in barley epidermal cells, transient-induced gene silencing (TIGS) technique developed by Schweizer et al. was used [30]. To generate the vector containing a partial sequence for suppression of *MLO* expression, the 12th exon region of the *Mlo* gene was amplified from Morex genomic DNA as a template using the primer pair (MLO-TIGS-F; 5’-CACCATGGGATCAAACATGAAGAGGTCC-3’/MLO-morex-R; 5’-TCATCCCTGGCTGAAGGAAAAATC-3’) and inserted into the pENTR-D/TOPO vector (Invitrogen, Carlsbad, CA, USA), resulting in pENTR-MLO-TIGS. Then, pANDA-mini-MLO was obtained by LR reaction with pANDA-mini vector, which is the Gateway vector for RNA interference [31]. The full-length cDNA of *MLO* was amplified from Morex leaf-derived cDNA synthesized as described by Yara et al. [32] using the primer pair (caccMLO-Morex-F; 5’-CACCATGTCGGACAAAAAAGGGGT-3’/MLOwo-R; 5’-TCCCTGGCTGAAGGAAAAATC-3’), and inserted into the pENTR-D/TOPO vector to generate pENTR-MLO. Then, pGWB5-MLO was obtained by LR reaction with pGWB5 vector. To observe the effects of TIGS in the long term with a minimum of stress, primary leaves attached to the living seedlings but not detached leaves were bombarded with gold particles coated with the mixture of pANDA-mini-MLO and pGWB5-MLO by using Helios Gene Gun System (Bio-Rad Laboratories, Hercules, CA, USA) as described by Wahara et al. [33]. After 5 days, GFP-expressing cells were counted using a fluorescent microscope (BX60, Olympus, Tokyo, Japan). As with pANDA-mini-MLO, the partial fragment of *MLA8* was amplified from pENTR-ubi-Mla8 as a template using the primer pair (MLA8RNAi-F2; 5’-CACCTTCCGCTGTATGGTTAACTTTCG-3’/MLA8RNAi-R2; 5’-TCAGTTCTCCTCATCATCAGAAAAATC-3’) and inserted into pENTR-D/TOPO vector to generate pENTR-MLA8-RNAi. Subsequently, pANDA-mini-MLA8 was obtained by LR reaction with pANDA-mini vector. TIGS constructs were co-bombarded with pENTR-ubi-GUS as an introduction marker to perform the penetration assay.

### Penetration assay on single cells

Approximately 35 h after inoculating conidia of OU14 and RACE1 isolates on the primary leaves bombarded with TIGS constructs, GUS staining was performed as described by Yaeno et al. [34] and *Bgh* was stained with Coomassie brilliant blue solution (0.3% w/v Coomassie brilliant blue R-250 [Nacalai Tesque, Kyoto, Japan], 7.5% trichloroacetic acid, and 30% methanol) and washed three times with water. The penetration rate was calculated by counting the number of conidia developing haustoria in GUS-expressing cells per total number of interactions between conidium and a GUS-expressing cell.

### Extraction of genomic DNA and sequencing of the *Mlo* gene

Genomic DNA was extracted from 7-day-old seedlings of *mlo* accessions as described in Kawamoto et al. [35] with slight modifications. Four cm^2^ leaf segments frozen with liquid nitrogen were powdered by bead crushing with Shake Master Neo (BMS-M10N21, Biomedical Science Co., Ltd., Tokyo, Japan). The DNA extraction buffer (1% (w/v) N-lauroyl sarcosyl sodium salt, 100 mM Tris, 100 mM NaCl, 0.32% (w/v) Na_2_EDTA, 2% (w/v) PVP, and 0.1 mg/mL RNase A) was added and incubated for 1 h at 37°C with vortexing every 20 min. The supernatant was extracted by PCI (phenol: chloroform: isoamyl alcohol = 25:24:1) and subsequent CI (chloroform: isoamyl alcohol = 1:1), and genomic DNA was obtained by 2-propanol precipitation and dissolved in Tris-EDTA buffer. Using primer pairs (MLO-F; 5’-GGCTGCTCCGCCAGCAAACCAGAC-3’/MLO1441-R; 5’-GTTGATGAAGCCTGCCCTCA-3’ and MLO1265-F; 5’-ACCCCTGGCATCAGATGGGTGG-3’/MLO-R; 5’-AGGGGTTTTGTTTGTGCTTAGCAG-3’), the *Mlo* gene region was amplified with PrimeSTAR MAX (Takara Bio Inc., Kusatsu, Shiga, Japan), resulting in fragments of approximately 1500 bp and 1900 bp, respectively, and sequenced by outsourcing to Eurofins Genomics (Tokyo, Japan). Multiple alignments of *Mlo* alleles were performed with GENETYX-MAC Ver. 20 (GENETYX Corp., Tokyo, Japan). Ten single nucleotide polymorphisms were found between these wild-type accessions that did not affect the function, which were classified as the Carlsberg II type (Carlsberg II, Haisa, and Malteria Heda) and the Pallas type (Pallas and Foma). P22 is a near-isogenic line of Pallas created using the *mlo*-5 mutant of Carlsberg II (R5678) and is therefore a Carlsberg II type [36,37].

## Results

### RACE1 can only penetrate the *mlo* mutant alleles that do not have *NLR* genes

**Fig 1:**
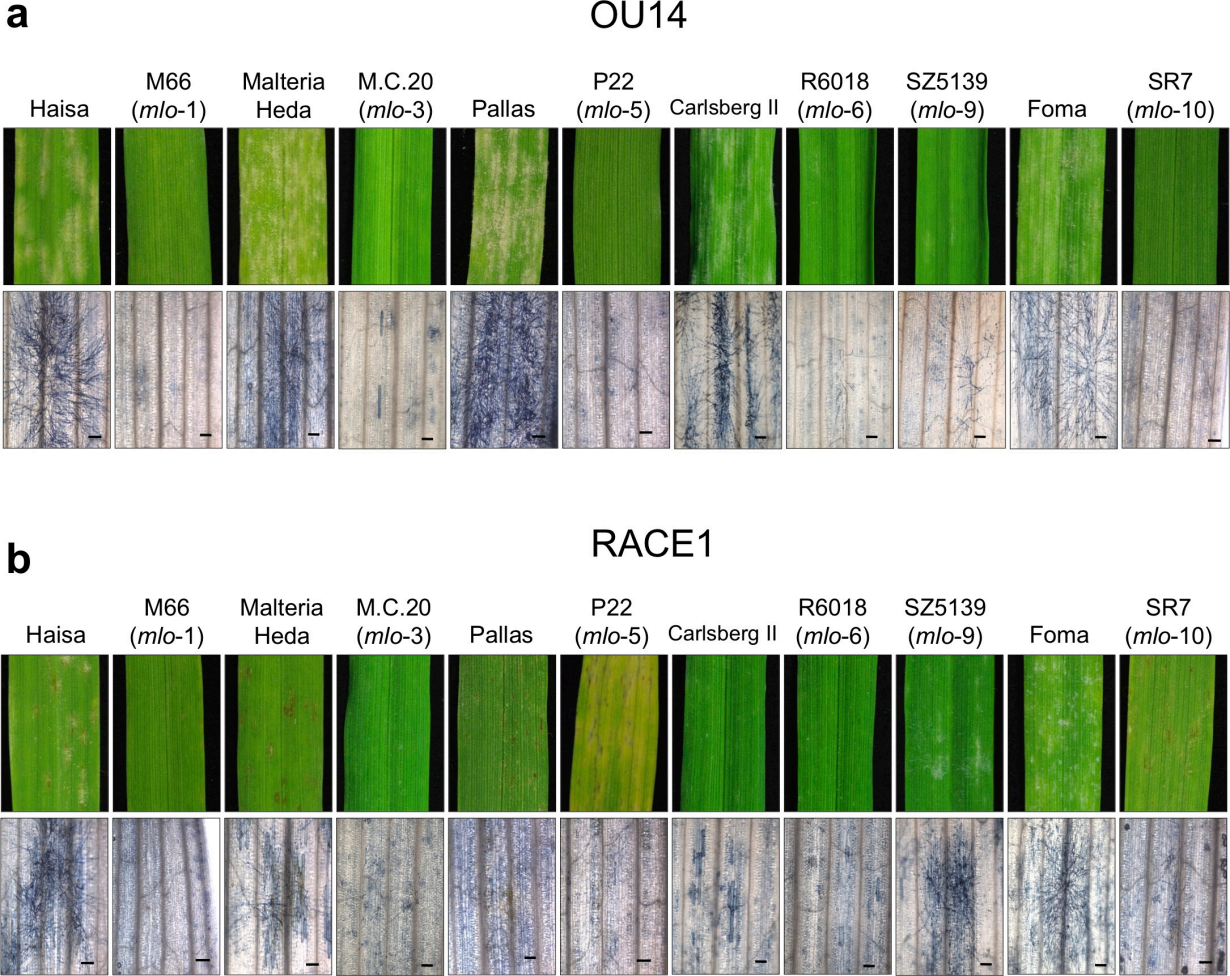
Visible and microscopic phenotypes of the *mlo* mutants inoculated with two different races of *Blumeria graminis* f. sp. *hordei*. Phenotypes of the primary leaves inoculated with OU14 (**a**) and RACE1 (**b**) are shown. To allow for natural inoculation, seedlings were grown for 10 to 14 days in chambers where each *Bgh* race was maintained. Microscopic observation after trypan blue staining was performed 4 days after inoculation. Scale bars indicate 0.2 mm.

**Fig 2:**
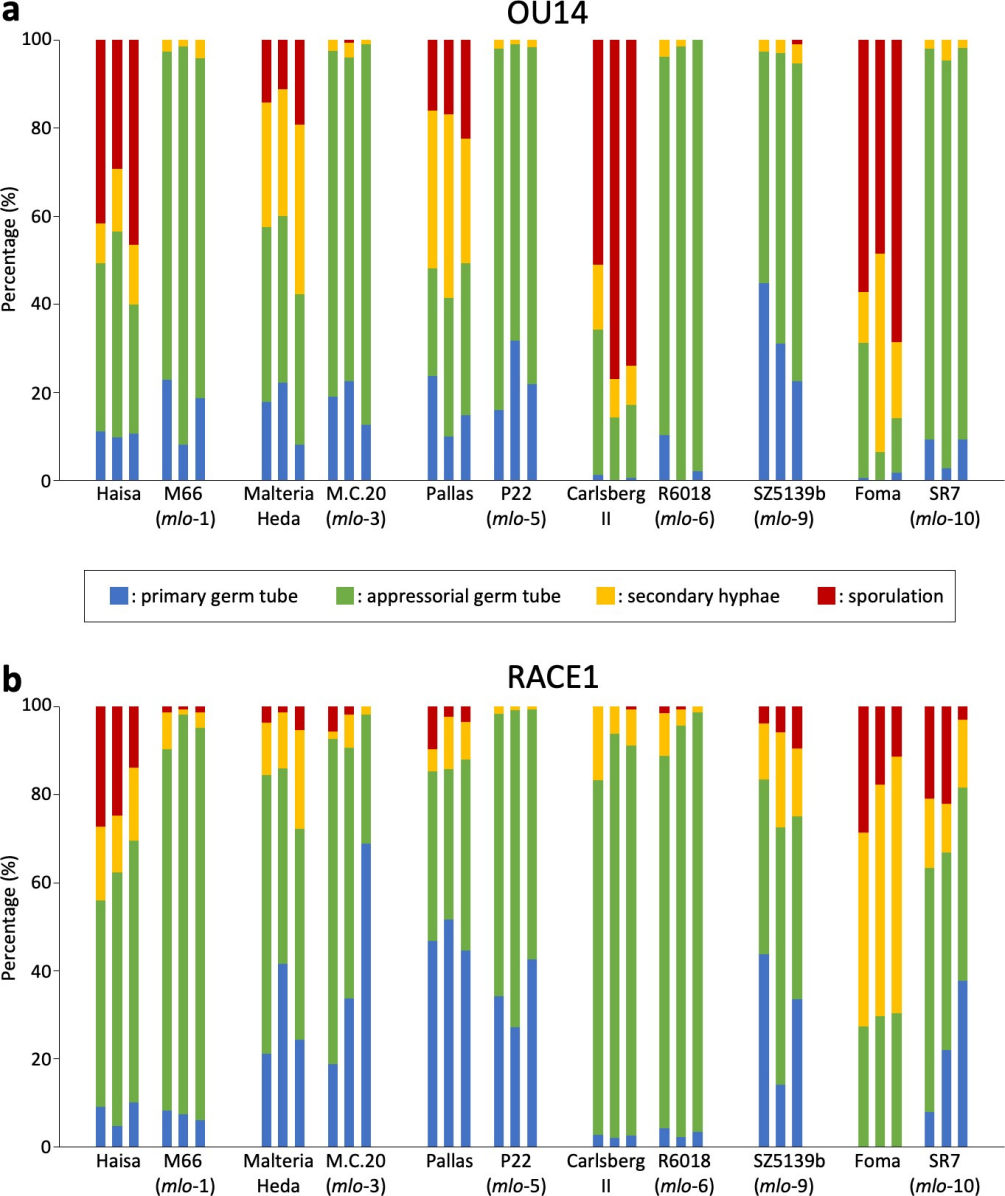
Proportion of the morphogenesis of *Blumeria graminis* f. sp. *hordei* races in the *mlo* mutants. The primary leaves 4 days after inoculation with OU14 (**a**) and RACE1 (**b**) were stained with trypan blue and the morphology of conidia in the situation where only one conidium was in contact with one epidermal cell was counted. The results of three independent experiments on the morphological proportions of about 100 to 200 conidia are shown. Blue indicates conidia arrested with the primary germ tube, green indicates conidia arrested with the appressorial germ tube, yellow indicates conidia arrested with the elongated secondary hyphae, and red indicates conidia that produced new conidia.

Similar to the report that the isolates of *Bgh* were selected by over 37 cycles of culture on the leaves of HLN70-8 derived from the *mlo*-9 mutant allele line SZ5139b [38], Lyngkjær et al. [26] had found that the Japanese isolate, RACE1, also overcame the *mlo*-mediated resistance in the mutant allele lines, M66 (*mlo*-1), M.C.20 (*mlo*-3), SR1 (*mlo*-4), SR7 (*mlo*-10), and Atem and Totem (both *mlo*-11). At that time, however, it was not clear which *NLR* gene each accession possessed, and it was not correctly interpreted because of the confusion between the broad-spectrum resistance by the *mlo* mutation and the race-specific resistance triggered by *NLR* genes. Even now, in many cases, it is not clear which *NLR* genes an accession possesses. Therefore, we decided to test whether RACE1 truly overcomes the *mlo*-mediated resistance by using the mutants and the corresponding wild-type accessions, which had been stocked at the Institute of Plant Science and Resources, Okayama University from 1994. Table 1 summarizes whether each of the wild-type accessions possesses an *NLR* gene against RACE1. Each line was grown for 10 to 14 days in chambers that maintained *Bgh* races. In the wild-type accessions, Haisa, Malteria Heda, and Foma, the sporulation of both OU14 and RACE1 was observed (Fig 1). However, RACE1 did not form conidia as abundantly as OU14, causing brownish-red necrotic patches. Although Haisa has been used as completely susceptible to European races, it was resistant to the Japanese race H14 [39]. Thus, this suggests that it has an unknown resistance gene and that the necrotic patches against RACE1 may be HR cell death caused by the resistance. It has been noted that Malteria Heda may possess either the allele *Mla6*, *Mla7*, or *Mla8* [40]. However, it was susceptible to OU14 harboring *AVRa6* and *AVRa7* and caused HR cell death against RACE1 harboring *AVRa6*, *AVRa7*, and *AVRa8*, suggesting that it possesses *Mla8*. Likewise, Pallas and Carlsberg II have *Mla8* [40,41]. Diamant, the wild-type accession of SZ5139b (*mlo*-9), is also resistant to RACE1 because it is an X-ray mutant of Valticky possessing *Mla8* [42]. On the other hand, Foma does not have *Mla8* or any other *Mla* alleles [43,44] and in fact, RACE1 was able to form conidia on it (Fig 1). Therefore, only the Foma-derived line, namely SR7, can be tested in this study to clarify whether RACE1 can infect the *mlo* mutants. OU14 was able to infect any wild-type accessions but failed to infect all *mlo* mutants without any symptoms. In contrast, RACE1, which could not infect most of the *mlo* mutants with *Mla8*, formed conidia while causing necrotic patches on the leaves of SR7 (*mlo*-10). Microscopic examination of *Bgh* revealed how far the infection has progressed (Fig 2). The primary leaf of healthy barley seedling was inoculated with *Bgh* and stained with trypan blue 4 days later. After attaching to the leaf surface and producing the primary germ tube, *Bgh* conidium produces the appressorial germ tube where the tip differentiates into the appressorium. If the penetration fails, the morphogenesis stops at the appressorial germ tube. Because the haustorium as a result of penetration is not easy to determine accurately under the microscope after trypan blue staining, the formation of secondary hyphae was used as an indicator of successful penetration. In some cases, even if the secondary hyphae are formed after penetration, its elongation is suppressed afterward. For this reason, sporulation was used as an indicator of complete infection. Similar to macroscopic observations, there were a number of conidia of OU14 that successfully penetrated and consequently sporulated in all wild types, but the majority of conidia were unable to form secondary hyphae and were arrested with the appressorial germ tubes in all *mlo* mutants, indicating an almost complete failure to penetrate (Fig 2A). In contrast, RACE1 was able to penetrate and sporulate in SR7 (*mlo*-10) (Fig 2B). These results suggest that RACE1 is capable of partially overcoming the *mlo*-10-mediated resistance.

**Table 1:** *NLR* gene (s) against *Blumeria graminis* f. sp. *hordei* isolate RACE1 in *mlo* mutant alleles.

Allele	Mutant ID	Wild-type accession	*NLR* gene	Reference
*mlo*-1	M66	Haisa	unknown *NLR*	[39], this study
*mlo*-3	M.C.20	Malteria Heda	*Mla8*	[40], this study
*mlo*-5	P22	Pallas	*Mla8*	[40]
*mlo*-6	R6018	Carlsberg II	*Mla8*	[40]
*mlo*-9	SZ5139	Diamant	*Mla8*	[42]
*mlo*-10	SR7	Foma	none	[43,44]

### The same genetic background should be used for the analysis of *mlo*-mediated resistance

**Fig 3:**
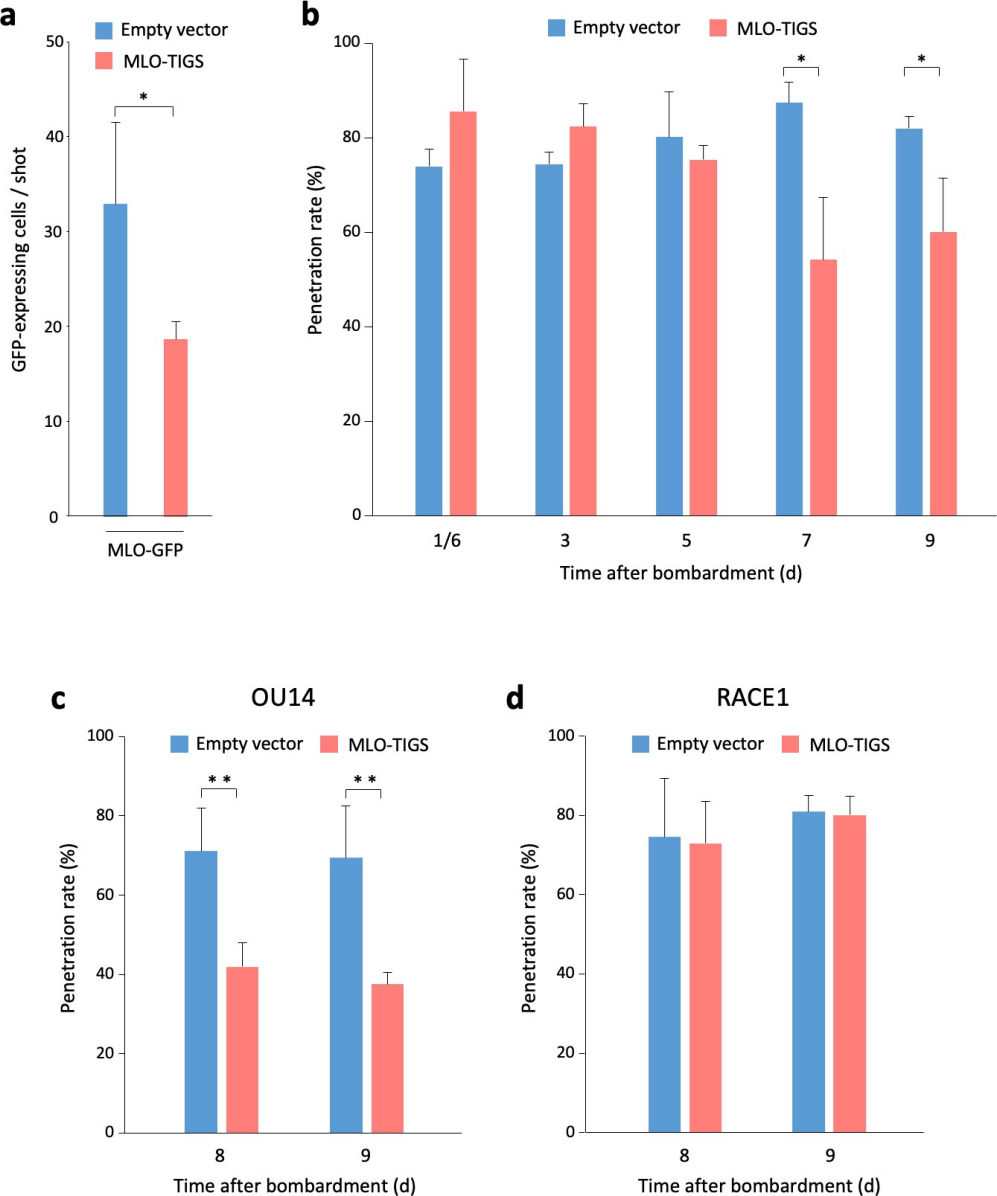
The penetration of OU14 but not RACE1 decreased in epidermal cells in which *MLO* expression was suppressed by transient induced gene silencing (TIGS). (**a**) Reduction of GFP-expressing cells by introducing MLO-TIGS construct. The number of Morex epidermal cells expressing GFP was counted 5 days after introduction with MLO-GFP and MLO-TIGS constructs. Error bars represent standard deviation (n = 5). (**b**) Penetration rate of OU14 decreased from 7 days after the introduction of MLO-TIGS construct. Seven-day-old primary leaves of Morex was introduced with MLO-TIGS and GUS constructs. Error bars represent standard deviation (n = 3). Penetration rates of OU14 (**c**) and RACE1 (**d**) in MLO-TIGS-introduced epidermal cells of six-day-old primary leaves of Morex. Younger seedlings were used to reduce the effects of senescence. Error bars represent standard deviation (n = 5). The empty pANDA-mini vector was introduced as a negative control. The mean values of these experiments were calculated from independent experimental data obtained from 2–10 introduced leaves. *, *P* < 0.05; **, *P* < 0.01 (Student’s *t*-test).

Since the rate of morphogenesis of RACE1 varied among accessions, it was difficult to understand whether the differential responses were derived from the *mlo* mutations, *Mla8*, or other unknown genes (Fig 2). For example, unlike other accessions with *Mla8*, in the Pallas lines, there was a high percentage of conidia that were arrested with the primary germ tube. This implies that there is a gene that may interfere with the formation of an appressorial germ tube. Besides, some lines, such as SZ5139b (*mlo*-9), were observed to be somewhat penetrated and sporulated, despite possessing *Mla8*. Hence, the same genetic background should be used when investigating the effects of the *mlo* mutation alone on the penetration of RACE1. In barley, only certain accessions, e.g. Golden Promise, can be efficiently transformed [45]. However, Golden Promise also possesses *Mla8* [40]. Therefore, using a susceptible accession, Morex, which does not possess any functional *Mla* gene, we investigated the penetration rate of RACE1 into cells in which the expression of the *MLO* was suppressed by transient-induced gene silencing (TIGS). Because *MLO* transcripts accumulate significantly in the first leaf even up to 3 weeks after sowing [46], it was necessary to inoculate RACE1 under conditions where the effects of TIGS last longer. In addition, the method of inoculating detached leaves reduces the penetration rate of *Bgh* to less than 20%, making it difficult to examine the effects of TIGS. Therefore, we used a single-cell expression system that allows long-term TIGS without detaching leaves [33]. First, to confirm whether TIGS works in epidermal cells of Morex leaves, both the construct expressing MLO fused with GFP at the C-terminus and the TIGS construct of *MLO* were simultaneously introduced by particle bombardment. The results showed that the number of GFP-expressing cells was significantly reduced when the TIGS construct was introduced compared to the empty vector. Therefore, the expression of *MLO-GFP* was confirmed to be suppressed by the simultaneously-introduced RNAi (Fig 3A). Since the suppression of *MLO* expression by the TIGS is not expected to immediately reduce the already accumulated protein in 7-day-old primary leaves, the penetration rates of OU14 after the introduction of the TIGS construct were examined to reveal the period of expression that could decrease the intrinsic MLO. As a result, the penetration rate of OU14 was significantly reduced from 7 days after introduction (Fig 3B). This suggests that the TIGS suppressed *de novo* protein synthesis and disrupted the protein turnover, resulting in a decrease in the level of MLO protein. The expression of *MLO* increases gradually by 14 days after sowing [46], but leaf tip senescence itself, which begins after 9 days, has a negative effect on the penetration of *Bgh*. Therefore, we examined the timing of a significant reduction in the penetration rate of OU14 after introduction into the youngest leaves available for introduction (6 days after sowing). As a result, the penetration rate of OU14 was significantly reduced 8 or 9 days after introduction (Fig 3C). On the other hand, however, the penetration rate of RACE1 remained high and unaffected by the TIGS (Fig 3D). These results demonstrated that RACE1 was able to overcome the penetration resistance conferred by the suppression of *MLO* expression, even in the same genetic background.

### RACE1 cannot penetrate the null mutant allele *mlo*-5

**Fig 4:**
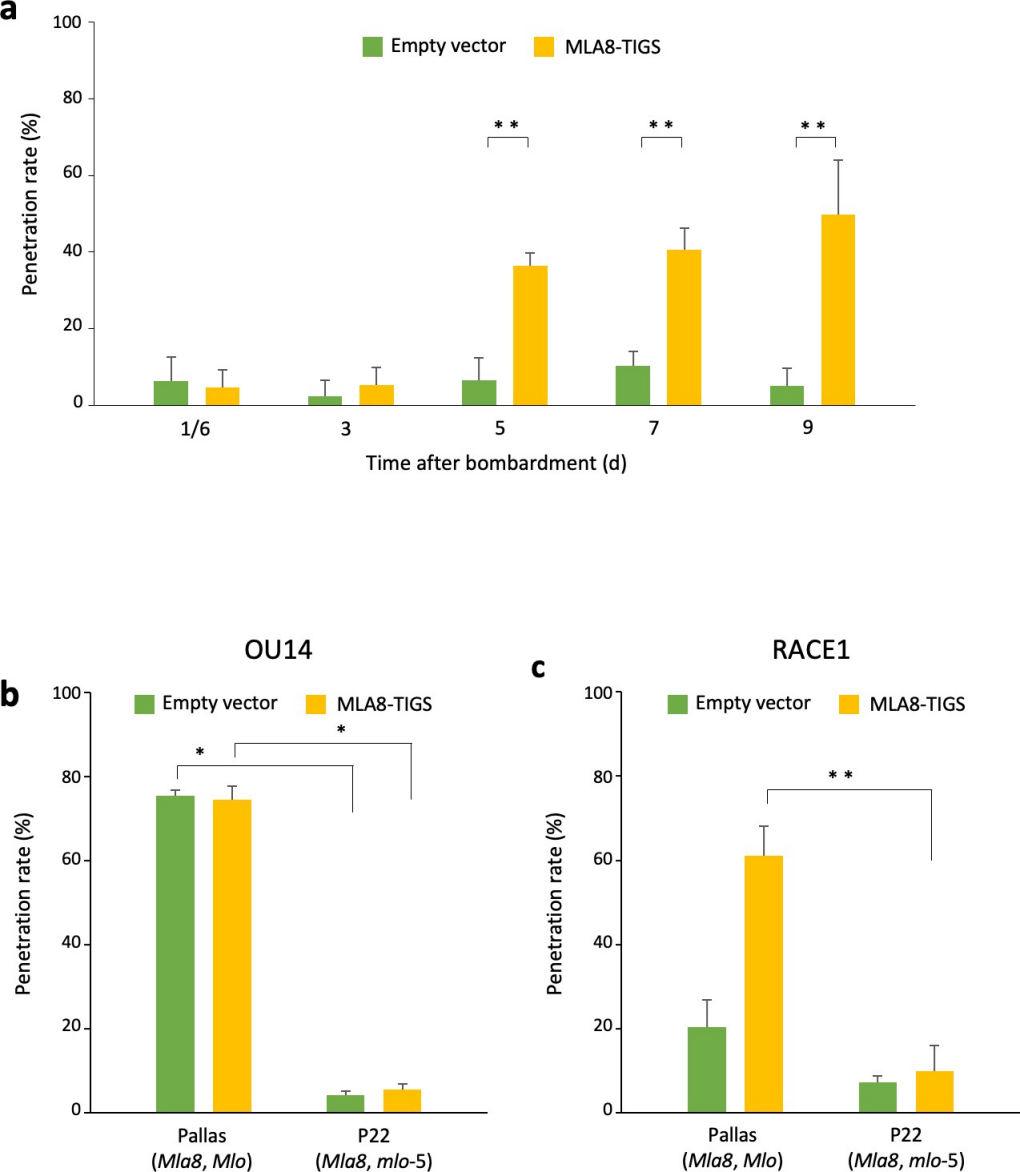
RACE1 was not able to overcome the penetration resistance conferred by the *mlo*-5 mutation. (**a**) Penetration rate of RACE1 increased from 5 days after the introduction of MLA8-TIGS construct in Pallas (*Mla8*) epidermal cells. Penetration rates of OU14 (**b**) and RACE1 (**c**) were examined in MLA8-TIGS-introduced epidermal cells of Pallas and P22 (*mlo*-5). The empty pANDA-mini vector was introduced as a negative control. Error bars represent standard deviation (n = 3). The mean values of these experiments were calculated from independent experimental data obtained from 2–10 introduced leaves. *, *P* < 0.05; **, *P* < 0.01 (Student’s *t*-test).

The reduction in the penetration rate of OU14 by the TIGS was less than half that in the *mlo* mutants (Figs 2A and 3C). It is unlikely that the expression of *MLO* is completely suppressed, and there may still be functional MLO remaining after the TIGS. Indeed, there is a supportive report that the strength of penetration resistance may differ depending on the amount of mutant protein accumulation, although the *mlo*-10 mutant has a similar level of protein accumulation to the wild type [47]. The *mlo*-mediated penetration resistance may be due to two changes: the loss of function and the reduced levels of MLO protein. With regard to the reduction of the protein levels, there may be a threshold at which OU14 cannot overcome while RACE1 can. If so, the penetration of RACE1 in the *mlo*-5 mutant, where no protein accumulates due to the mutated initiation codon, needs to be investigated. Therefore, the expression of *MLA8* was suppressed by the TIGS to investigate the penetration rate of RACE1 in Pallas 22 (P22; *mlo*-5). As in the case of the TIGS of *Mlo*, we investigated the timing of the effects of the TIGS of *MLA8* and found that the penetration rate of RACE1 in Pallas continued to increase significantly from 5 to 9 days after introduction (Fig 4A). Based on the results, we next investigate the penetration rate of OU14 and RACE1 in the P22 9 days after the introduction of the TIGS construct of *MLA8*. Regardless of the suppression of *MLA8* expression, OU14 failed to penetrate P22 (Fig 4B). Similarly, even with *MLA8* suppression, RACE1 was almost unable to penetrate P22 (Fig 4C). RACE1, which doesn’t harbor *AVRa1* and *AVRa22* [10,11], could sporulate in near-isogenic Pallas lines possessing *Mla1* or *Mla22*, indicating that Pallas possesses no *NLR* locus against RACE1 other than on the *Mla* locus (S1 Fig). These results suggest that RACE1 is unable to overcome the penetration resistance once the *mlo*-5 mutation prevents the synthesis of the MLO protein.

### RACE1 can overcome the *mlo*-mediated resistance in the transformable Golden Promise with suppression of *MLA8*

**Fig 5:**
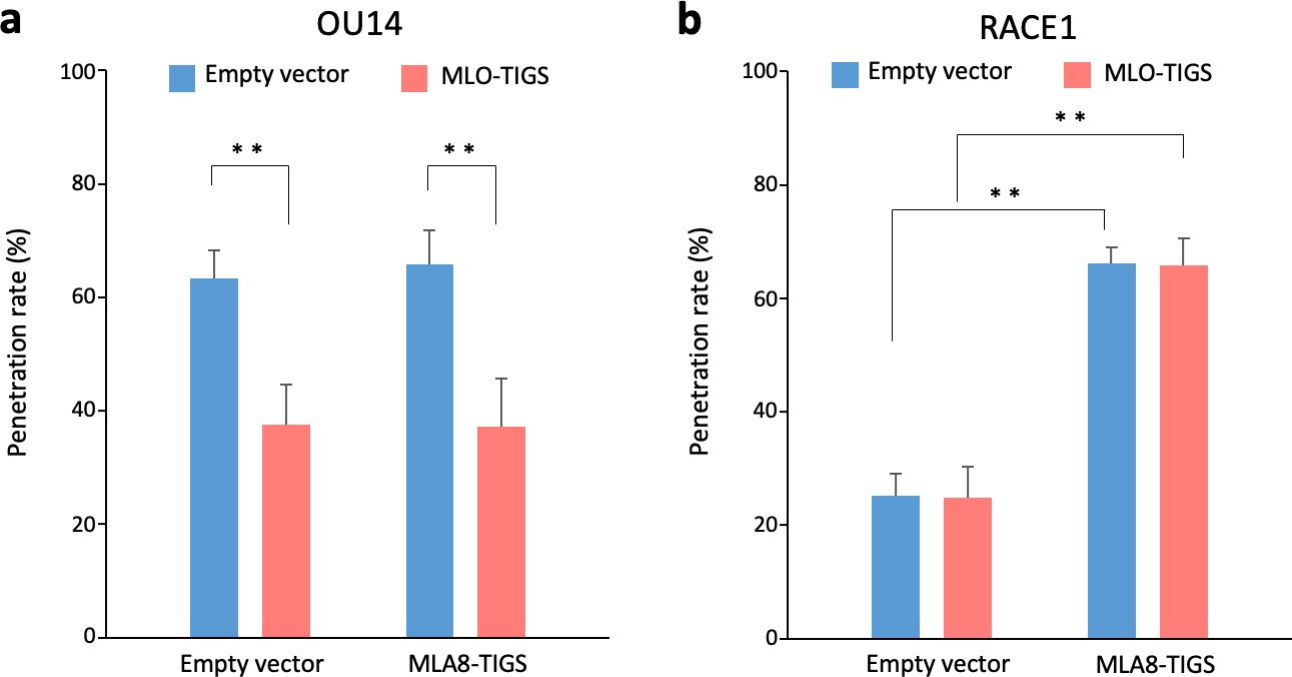
RACE1 overcome the penetration resistance in Golden Promise epidermal cells in which *MLO* and *MLA8* expression was suppressed. Penetration rates of OU14 (**a**) and RACE1 (**b**) were examined in MLO-TIGS- and MLA8-TIGS-introduced epidermal cells of Golden Promise (*Mla8*). The empty pANDA-mini vector was introduced as a negative control. Error bars represent standard deviation (n = 3). The mean values of these experiments were calculated from independent experimental data obtained from 2–10 introduced leaves. **, *P* < 0.01 (Student’s *t*-test).

To elucidate the mechanism by which RACE1 overcomes the *mlo*-mediated penetration resistance, the correlation with allele-dependent protein levels will need to be analyzed. Since the quantification of protein levels in single cells in which the *MLO* expression is suppressed by the TIGS is technically very difficult, generating lines with a variety of alleles using genome editing techniques would be a useful approach. Before it can be undertaken in the future, it must first be confirmed that RACE1 can overcome the *mlo*-mediated penetration resistance in the transformable Golden Promise. The penetration rates of both races were compared when *MLO* and *MLA8* expression was simultaneously suppressed (Fig 5). The penetration rate of OU14 was reduced when *MLO* expression was suppressed, regardless of *MLA8* suppression. The penetration of RACE1 was suppressed when *MLA8* expression was not suppressed. In contrast, the suppression of *MLA8* expression allowed RACE1 penetration even with the suppression of *MLO* expression. These results indicate that RACE1 can overcome the *mlo*-mediated penetration resistance even in Golden Promise when *MLA8* is absent. Furthermore, the use of *mlo* allele-introduced lines of Golden Promise, which lacks both *Mla8* and *Mlo*, will allow us to elucidate the relationship between allele-dependent MLO protein levels and the breaking of the penetration resistance by RACE1.

## Discussion

In this study, it was demonstrated that RACE1 isolate overcomes partially the penetration resistance conferred by the *mlo*-10 mutation or the suppression of *Mlo* expression. Although most *mlo* accessions have been bred with *NLR* genes such as *Mla8* and therefore the spread of RACE1 infection would not occur, it should be noted that a mutation in the corresponding *Avr* gene would be possible. The *mlo*-5 mutation, which cannot be overcome by RACE1, appears to be an effective genetic resource but has not been used because it tends to cause senescence [46]. In fact, the leaves turned yellow severely with HR cell death when P22 was inoculated with RACE1, although the resistance responses triggered by *Mla8* may have accelerated senescence. Lyngkjær et al. [26] showed that RACE1 formed a moderate number of colonies in both the M66 (*mlo*-1 mutant in Haisa) and the M.C.20 (*mlo*-3 mutant in Malteria Heda). However, in our M66 and M.C.20 lines, in which the *mlo*-1 and *mlo*-3 mutations were identified by sequencing, respectively, the proportion of sporulating RACE1 was less than 10% (Fig 2), making it difficult to state that RACE1 was moderately infectious. RACE1 did not sporulate in the *mlo*-5 null mutant but did sporulate slightly in *mlo*-1 and *mlo*-3. This fact implies a possibility that the few remaining mutant proteins allow RACE1 to overcome slightly the resistance. This possibility can only be investigated in accessions in which there are no *NLR* genes recognizing RACE1. Lyngkjær et al. [26] also reported that RACE1 partially infects the *mlo*-11 mutant accessions Atem and Totem, but not several *mlo*-11 lines other than Atem and Totem. It might need to be validated whether these accessions possess other *NLR* genes or whether the accessions used really possess the *mlo*-11 mutation. In fact, Reinstädler et al. [48] reported that one of eight “*mlo*-11 mutant alleles” tested was not *mlo*-11. Recently, it was also reported that about 85% of accessions of the core collection of the Czech winter barley gene bank was heterogenous [49]. In addition, we found that the wild-type Diamant seeds were suspected to be heterogenous due to contamination with other *mlo* mutant alleles, and avoided using them. Hence, the sequences of the *Mlo* gene regions of all the lines used in this study were confirmed (S2 Fig). Taken together, the accessions with unknown genetic backgrounds should be carefully analyzed. It would be also reasonable to use lines with the same background if molecular genetic techniques such as TIGS are available.

The suppression of *MLO* expression by the TIGS was insufficient to suppress the penetration of OU14, suggesting that the intrinsic MLO protein was not completely reduced in abundance after 9 days of introduction (Fig 5). In other words, this means that even OU14 can penetrate if a certain amount of MLO protein is present. Indeed, Müller et al. [47] reported that mutant MLO proteins accumulated moderately in SZ5139b (*mlo*-9) and at wild-type levels in SR7 (*mlo*-10), but very low levels in M66 (*mlo*-1). Unlike OU14, RACE1 was able to sporulate to some extent in SZ5139b and SR7, suggesting a correlation between MLO protein levels and its ability to overcome the penetration resistance. To examine the possibility that such a threshold of protein levels exists, it would be necessary to compare the effects of alleles in the same genetic background using a stably transformable accession, i.e., Golden Promise. For example, the complementary introduction of each *mlo* allele with an epitope tag into Golden Promise, which has lost both *Mla8* and *Mlo* by genome editing techniques, will allow for a detailed analysis of the correlation between the protein levels and the penetration resistance against RACE1. In addition, they may be powerful tools for solving the trade-off problems among other disease responses and symbiosis. Barley *mlo* mutants are hyper-sensitive to the rice blast fungus, *Magnaporthe oryzae* [50]. On the other hand, the symbiotic performance of the arbuscular mycorrhizal fungi *Funneliformis mosseae* and *Rhizophagus irregularis* is reduced in the *mlo* mutants [51,52]. Thus, the identification of *mlo* alleles that show a certain level of resistance to *Bgh*, such as RACE1, as well as rice blast, and that are not incompatible with the symbiotic fungi may provide a clue to solving the trade-off problem by controlling the protein level of MLO.

In this study, it was found that RACE1 partially overcomes the *mlo*-mediated penetration resistance, albeit in an allele-dependent manner. However, the mechanism is unknown because the biochemical activity of MLO itself is not yet known. The genome structure of RACE1, which is significantly different from other races, may give rise to specialized effectors that can suppress the resistance [10]. Identifying the effectors involved in the suppression and uncovering part of the mechanism by which RACE1 overcomes the resistance would also help to elucidate the molecular function of MLO.

## Supporting information

S1 Fig Pallas possesses no *NLR* locus against RACE1 other than on the *Mla* locus. Primary leaves of near-isogenic Pallas lines *Mla1* or *Mla22* were inoculated with OU14 and RACE1. Both isolates could infect P12 (*Mla22*), indicating that Pallas doesn’t possess no *NLR* genes against them other than *Mla8*.
(TIF)

S2 Fig Genomic sequences of *Mlo* gene region cloned from the *mlo* mutant alleles and the wild-type accessions. The multiple alignments of only the regions where the *mlo* mutations and the single nucleotide polymorphisms (SNPs) exist are shown. The black lines indicate partial regions of each exon. Asterisks indicate SNPs.
(TIF)
