# Supplementary figures and images for: RACE1, a Japanese Blumeria graminis f. sp. hordei isolate, is capable of overcoming partially mlo-mediated penetration resistance in barley in an allele-specific manner
Source: PLoS One. 2021 Aug 23;16(8):e0256574. doi: 10.1371/journal.pone.0256574 (PMC8382181; doi:10.1371/journal.pone.0256574)

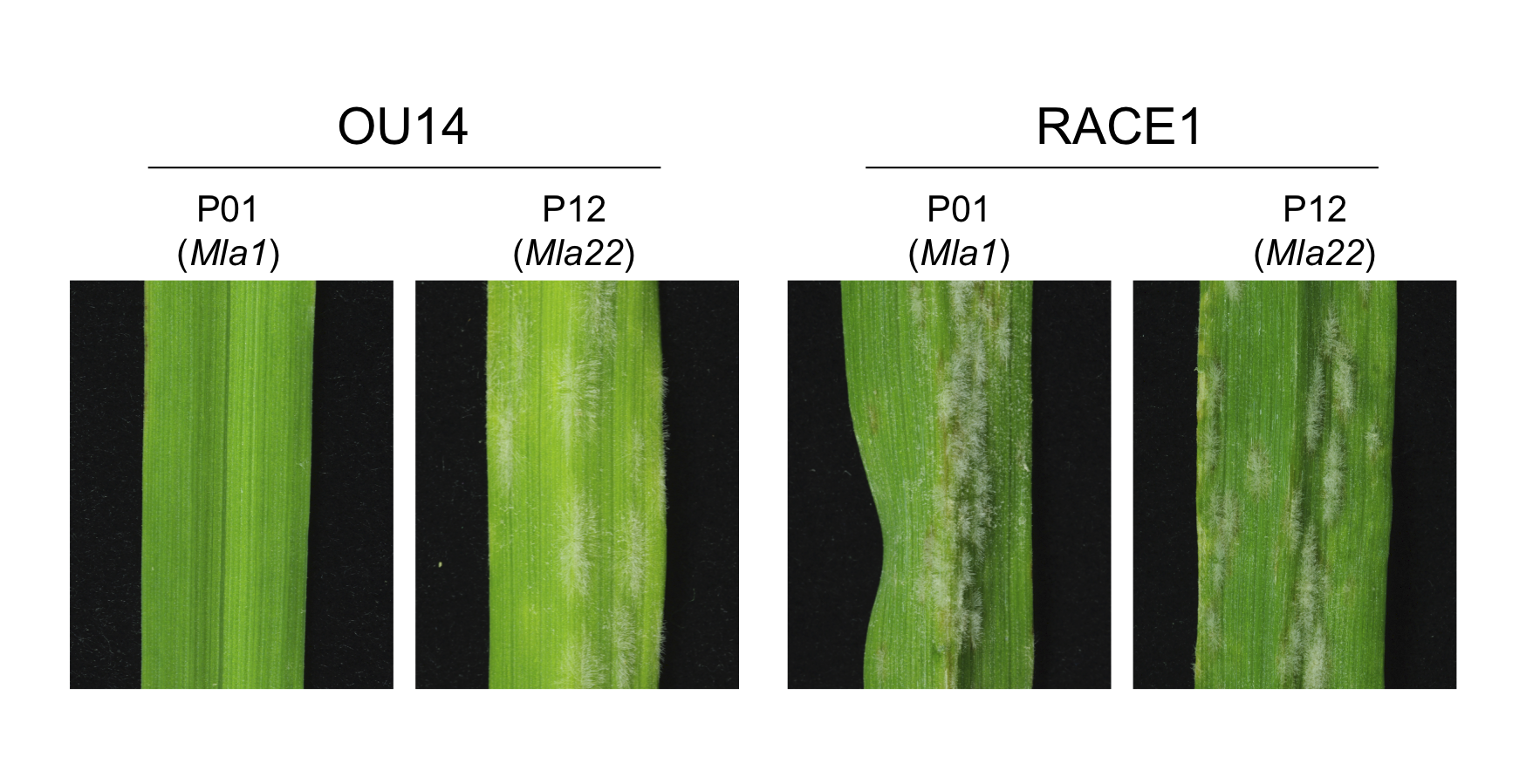

Supplement: S1 Fig — Primary leaves of near-isogenic Pallas lines Mla1 or Mla22 were inoculated with OU14 and RACE1. Both isolates could infect P12 (Mla22), indicating that Pallas doesn’t possess no NLR genes against them other than Mla8. (TIF) [file pone.0256574.s001.tif]

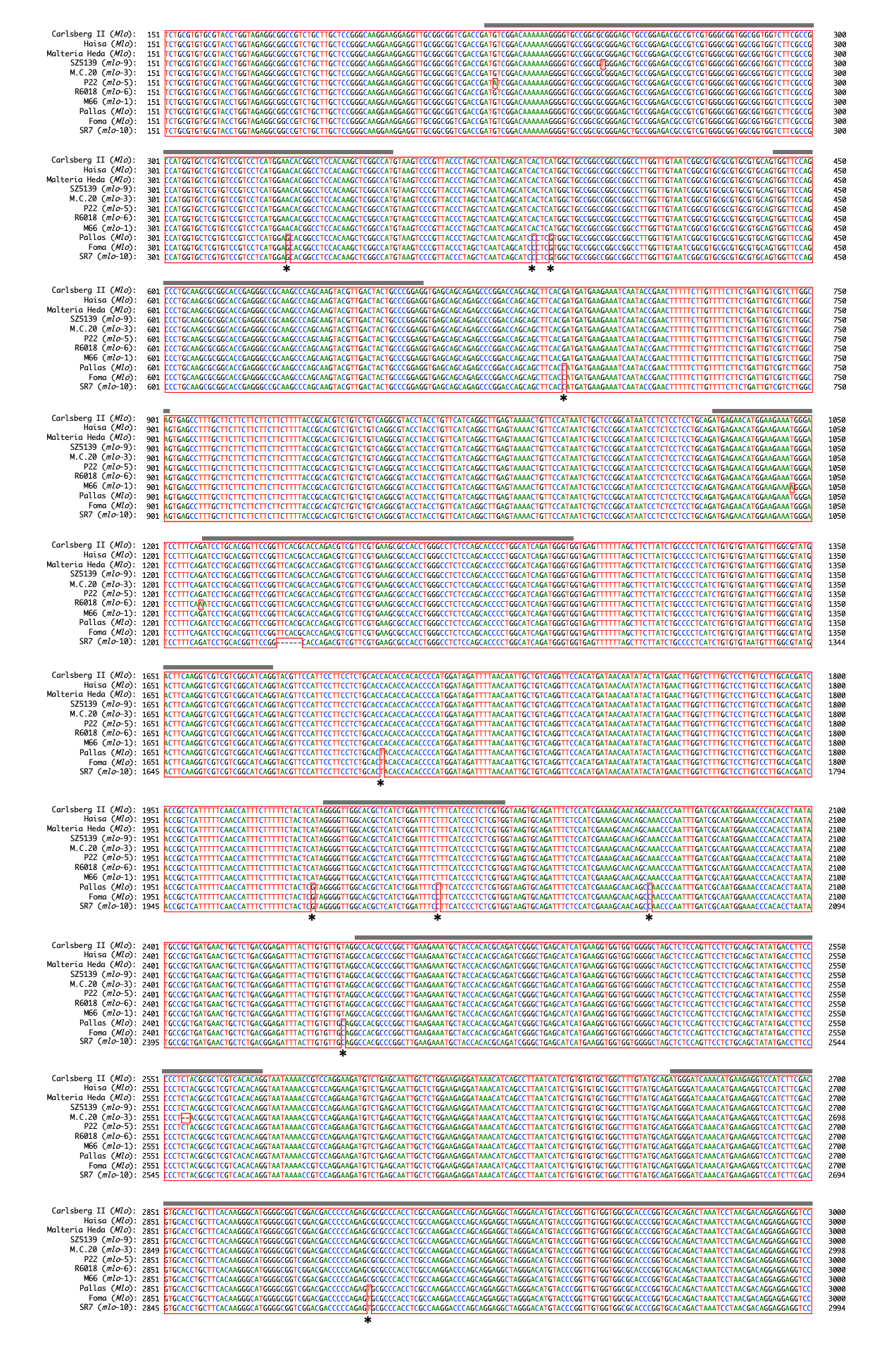

Supplement: S2 Fig — The multiple alignments of only the regions where the mlo mutations and the single nucleotide polymorphisms (SNPs) exist are shown. The black lines indicate partial regions of each exon. Asterisks indicate SNPs. (TIF) [file pone.0256574.s002.tif]
